# Supplementary material for: Ethnic differences in respiratory disease for Native Hawaiians and Pacific Islanders: Analysis of mediation processes in two community samples
Source: PLoS One. 2023 Aug 25;18(8):e0290794. doi: 10.1371/journal.pone.0290794 (PMC10456168; doi:10.1371/journal.pone.0290794)
Supplement: S1 Table — (DOCX) [file pone.0290794.s001.docx]

| Variable | Age | Sex | Educ | NH | Fil | PI | White |
| --- | --- | --- | --- | --- | --- | --- | --- |
| Age | -- | -.04 | .12 | -.13 | -.08 | -.15 | .16 |
| Sex | -.03 | -- | -.05 | -.03 | -.03 | .00 | .07 |
| Education | .11 | -.05 | -- | -.21 | -.09 | -.13 | .20 |
| Native Hawaiian | -.11 | -.05 | -.18 | -- | -.20^a^ | -.14^a^ | -.43^a^ |
| Filipino | -.12 | -.01 | -.11 | -.16^a^ | -- | -.10^a^ | -.32^a^ |
| Pacific Islander | -.12 | .02 | -.10 | -.07^a^ | -.06^a^ | -- | -.21^a^ |
| White | .14 | .04 | .09 | -.37^a^ | -.35^a^ | -.14^a^ | -- |
|  |  |  |  |  |  |  |  |
