# Supplementary material for: Ethnic differences in respiratory disease for Native Hawaiians and Pacific Islanders: Analysis of mediation processes in two community samples
Source: PLoS One. 2023 Aug 25;18(8):e0290794. doi: 10.1371/journal.pone.0290794 (PMC10456168; doi:10.1371/journal.pone.0290794)
Supplement: S2 Table — (DOCX) [file pone.0290794.s002.docx]

| Variable | E-cig | Cig | Fin | SHS | BMI |
| --- | --- | --- | --- | --- | --- |
| E-cigarette use | -- | .52 | n.a. | .36 | .00 |
| Cigarette smoking | .59 | -- | n.a. | .47 | -.01 |
| Financial stress | .16 | .14 | -- | n.a. | n.a. |
| SHS exposure | .37 | .47 | .20 | -- | -.02 |
| BMI | .03 | .03 | .07 | .01 | -- |
|  |  |  |  |  |  |
