# Supplementary material for: Ethnic differences in respiratory disease for Native Hawaiians and Pacific Islanders: Analysis of mediation processes in two community samples
Source: PLoS One. 2023 Aug 25;18(8):e0290794. doi: 10.1371/journal.pone.0290794 (PMC10456168; doi:10.1371/journal.pone.0290794)
Supplement: S3 Table — (DOCX) [file pone.0290794.s003.docx]

| 2016 DATA |  |  |
| --- | --- | --- |
| Indirect effect | Critical ratio | p |
| Education=>E-cigarette use => Asthma | -2.64 | < .01 |
| Education -> Financial stress => Asthma | -3.44 | < .001 |
| Education => BMI => Asthma | -3.43 | < .001 |
| Education => Cigarette smoking => COPD | -6.47 | < .0001 |
| Education => Financial stress => COPD | -3.63 | .0001 |
| Education => SHS exposure => COPD | -2.86 | < .01 |
|  |  |  |
| 2018 DATA |  |  |
| Education => E-cigarette use => Asthma | -2.92 | < .01 |
| Education => SHS exposure => Asthma | -2.97 | < .01 |
| Education => BMI => Asthma | -3.45 | < .001 |
| Education => E-cigarette use => COPD | -2.82 | < .01 |
| Education => Cigarette smoking => COPD | -3.35 | < .001 |
| Education => SHS exposure => COPD | -2.41 | .01 |
| Education => BMI => COPD | -2.80 | < .01 |
|  |  |  |
|  |  |  |
